# *Fusobacterium nucleatum* exacerbates chronic obstructive pulmonary disease in elastase‐induced emphysematous mice

**DOI:** 10.1002/2211-5463.13369

**Published:** 2022-01-30

**Authors:** Ryuta Suzuki, Noriaki Kamio, Tadayoshi Kaneko, Yoshiyuki Yonehara, Kenichi Imai

**Affiliations:** ^1^ Department of Oral and Maxillofacial Surgery II Nihon University School of Dentistry Tokyo Japan; ^2^ Department of Microbiology Nihon University School of Dentistry Tokyo Japan

**Keywords:** chronic obstructive pulmonary disease, COPD exacerbations, emphysema, *Fusobacterium nucleatum*, periodontal disease

## Abstract

Exacerbation of chronic obstructive pulmonary disease (COPD) is associated with disease progression and increased mortality. Periodontal disease is a risk factor for exacerbation of COPD, but little is known about the role of periodontopathic bacteria in this process. Here, we investigated the effects of intratracheal administration of *Fusobacterium nucleatum*, a periodontopathic bacteria species, on COPD exacerbation in elastase‐induced emphysematous mice. The administration of *F. nucleatum* to elastase‐treated mice enhanced inflammatory responses, production of alveolar wall destruction factors, progression of emphysema, and recruitment of mucin, all of which are symptoms observed in patients with COPD exacerbation. Hence, we propose that *F. nucleatum* may play a role in exacerbation of COPD.

## Graphical abstract

Periodontal disease is a risk factor for chronic obstructive pulmonary disease (COPD) exacerbations. We found that intratracheally administered *Fusobacterium nucleatum*, a periodontopathic bacterium that increases in saliva when periodontal disease advances, to elastase‐induced emphysematous mice enhances (i) inflammatory responses, (ii) alveolar wall destruction factor production, (iii) emphysema progression, and (iv) mucin hypersecretion. These are known symptoms observed in patients with COPD exacerbations.

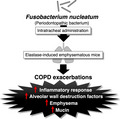

**Abbreviations**
AB/PAS: Alcian blue/Periodic acid‐Schiff
BALF: bronchoalveolar lavage fluid
BHI: brain‐heart infusion
CFU: colony‐forming units
COPD: chronic obstructive pulmonary disease
Lm: mean linear intercept
MMP: matrix metalloproteinase
MUC5AC: mucin 5AC
PBS: phosphate‐buffered saline

Chronic obstructive pulmonary disease (COPD), the third leading cause of mortality worldwide, is characterized by emphysema and limitation of the airflow, symptoms that are likely result from chronic inflammation in the lung periphery [1, 2]. COPD is also characterized by progression, which occurs by the repetition of COPD exacerbations [3]. Repeated exacerbations of COPD, which is frequently caused by bacterial or viral infection [4], are associated with worsened lung function and increased risk of death [3]. COPD exacerbation leads to increased infiltration of inflammatory cells [5, 6] and production of proinflammatory cytokines [7, 8] and proteases, such as matrix metalloproteinases (MMPs) [9], in the lungs of patients. Repeated COPD exacerbations accelerate the progression of emphysema [10] and induce hypersecretion of mucus [11]. Therefore, effective prevention of COPD exacerbations has important implications for clinical management and public health.

Periodontal disease, one of the most prevalent diseases worldwide, is an inflammatory reaction induced by infection by bacteria, such as *Porphyromonas gingivalis* and *Fusobacterium nucleatum*. It results in the destruction of the periodontium, including the periodontal bone, leading to tooth loss [12, 13]. Recently, several studies have found that periodontal disease is a risk factor for several systemic diseases, such as pneumonia, diabetes, and atherosclerosis [12, 14]. Epidemiological evidence indicates that periodontal disease is also associated with exacerbations of COPD [15, 16]. A systematic review indicated that periodontal treatment in patients with COPD and periodontal disease is associated with reduction in the frequency of COPD exacerbation and a slower rate of decline of lung function [17]. The European Federation of Periodontology and the American Academy of Periodontology have proposed that aspiration of dental plaque and/or hematogenous dissemination of inflammatory mediators from periodontal pockets underlie the worsening of COPD inflammatory status [18]. However, the details of the relationship between periodontal disease and COPD are not fully understood.

*Fusobacterium* *nucleatum*, a Gram‐negative anaerobe, is abundant in the oral cavity and has been implicated in various forms of periodontal disease, including a mild reversible form of gingivitis and advanced irreversible forms of periodontitis, including chronic periodontitis [19]. Recent reports have indicated that *F. nucleatum* is associated with several systemic diseases, including respiratory diseases such as pneumonia [19] and COPD [20], colorectal cancer [21], and gastric cancer [22]. In patients with severe COPD, the number of members of the genus *Fusobacterium* was found to be increased in bronchoalveolar lavage fluid (BALF) [23] and sputum [24]. Previously, we demonstrated that heat‐killed *F. nucleatum* strongly induces proinflammatory cytokines in primary respiratory epithelial cells and respiratory epithelial cell lines [25, 26]. Therefore, it is quite possible that tracheal aspiration of *F. nucleatum* plays a role in COPD exacerbations. Despite its importance, the relationship between oral bacteria, including *F. nucleatum*, and COPD exacerbation remains unexamined, and it is not clear whether this relationship is causal. In this study, we investigated the effects of intratracheal administration of heat‐killed *F. nucleatum* on the exacerbation of COPD in elastase‐induced emphysematous mice. Because *F. nucleatum*, an anaerobic bacterium, is considered to be incapable of survival in the respiratory tract, we used heat‐killed *F. nucleatum*. We found that administration of *F. nucleatum* to elastase‐treated mice enhanced the inflammatory responses, production of alveolar wall destruction factors, recruitment of mucin, and progression of emphysema.

## Materials and methods

### Preparation of bacteria

*Fusobacterium* *nucleatum* ATCC 25586 was cultured in brain–heart infusion (BHI) broth supplemented with 5 µg·mL^−1^ hemin and 0.5 µg·mL^−1^ menadione. The bacterial cell culture was incubated at 37 °C for 24–72 h in an anaerobic chamber (TE‐HERANAEROBOX, Hirasawa, Tokyo, Japan) under aerobic conditions of 10% CO_2_, 10% H_2_, and 80% N_2_. The bacterial cells were centrifuged at 7000 ***g*** for 10 min at 4 °C, and the pellet was resuspended in phosphate‐buffered saline (PBS). The bacterial cell density was adjusted to 1.0 × 10^10^ colony‐forming units (CFU)·mL^−1^. The bacterial suspension was heat‐killed at 60 °C for 1 h and then stored at −80 °C until use.

### Mice

Eight‐week‐old male C57BL/6J mice were purchased from CLEA Japan (Tokyo, Japan). All mice had free access to food and water and were kept in temperature‐controlled room (23 °C) on reverse 12/12 h light/dark cycle. All experimental protocols and procedures were performed in compliance with the Nihon University Rules concerning Animal Care and Use. All animal experiments were approved by the Nihon University Animal Care and Use Committee (AP18DEN031). The mice were euthanized by CO_2_ asphyxiation before harvesting lung specimens or BALF.

### Elastase‐induced emphysema mouse model

Mice under isoflurane anesthesia were intratracheally injected with 5 U of porcine pancreatic elastase (Elastin Products, Owensville, MO, USA). PBS‐treated age‐matched mice were used as controls. Three weeks after elastase or PBS injection, the mice under isoflurane anesthesia were intratracheally administered either 1 × 10^8^ CFU of *F. nucleatum* suspended in 50 µL of PBS or PBS alone every day for 7 days.

### Morphometric measurements of air space size

To measure the air space size, lung specimens were harvested early (1, 3, and 7 days) and late (42 days) after the last *F. nucleatum* administration and were fixed with 4% paraformaldehyde, embedded in paraffin, cut out as thin sections with 4 µm thickness, and stained with hematoxylin and eosin [27]. The linear intercepts of 100 alveoli were measured, and the mean linear intercept (Lm) was used as a morphometric parameter of emphysema [27, 28].

### Collection and analysis of bronchoalveolar lavage fluid

At 1, 3, and 7 days after the last *F. nucleatum* administration, whole lungs were washed three times with 1 mL of PBS, and BALF was collected from each mouse. The number of cells in the BALF was determined using a hemocytometer. Cell differentials were counted on smears prepared using a Cytospin and stained with Giemsa stain (Muto Pure Chemicals, Tokyo, Japan). The number of cells in each inflammatory cell fraction found in each BALF was estimated by multiplying the total number of cells that had been counted with a hemocytometer by the ratio of each cell fraction per 100 cells in the smear.

### Real‐time quantitative PCR

Total RNA was isolated from whole lungs using RNeasy Plus Mini Kits (Qiagen, Hiden, Germany) according to the manufacturer’s instructions. Synthesis of cDNA from total RNA was performed using PrimeScript RT Master Mix (Takara Bio, Shiga, Japan). The following primers were used in this study: *Mmp12*, forward 5′‐TGGTATTCAAGGAGATGCACATTT‐3′ and reverse 5′‐ GGTTTGTGCCTTGAAAACTTTTAGT‐3′; *Tnf*, forward 5′‐CTGTGCTCAGAGCTTTCAACAACTA‐3′ and reverse 5′‐TCCTTGATGGTGGTGCATGA‐3′; *Il6*, forward 5′‐GAGGATACCACTCCCAACAGACC‐3′ and reverse 5′‐AAGTGCATCATCGTTGTTCATACA‐3′; *Cxcl1*, forward 5′‐TGTGGGAGGCTGTGTTTGTA‐3′ and reverse 5’‐ACGAGACCAGGAGAAACAGG‐3′; *Cxcl5*, forward 5′‐GGTCCACAGTGCCCTACG‐3′ and reverse 5′‐GCGAGTGCATTCCGCTTA‐3′; *Ccl2*, forward 5′‐TTAAAAACCTGGATCGGAACCAA‐3′ and reverse 5′‐GCATTAGCTTCAGATTTACGGGT‐3′; *Cxcl10*, forward 5′‐ACCCAAGTGCTGCCGTCATT‐3′ and reverse 5′‐ATTCTCACTGGCCCGTCATC‐3′; *Muc5ac*, forward 5′‐CATGGAGGGGACCTGGAAAC‐3′ and reverse 5’‐ CCACACTGGGGTCACACTTC‐3′; *Actb*, forward 5′‐GGTCAGAAGGACTCCTATGTGG‐3′ and reverse 5′‐TGTCGTCCCAGTTGGTAACA‐3′. The amplification and detection of the cDNA was accomplished using a TP‐800 Thermal Cycler Dice Real‐Time System (Takara Bio) with TB Green Premix Ex Taq (Takara Bio).

### Western Blotting

Lung tissue extracts and BALF supernatants were prepared, separated using SDS/PAGE, and transferred to PVDF membranes. The membranes were blocked with 2% BSA and incubated with primary antibodies overnight at 4 °C. The primary antibodies included anti‐MMP12 (1 : 1000; Proteintech, Rosemont, IL, USA), anti‐perforin (1 : 1000; Cell Signaling Technology, Danvers, MA, USA), and anti‐β‐actin antibodies (1 : 200; Santa Cruz Biotechnology, Santa Cruz, CA, USA). The membranes were washed with Tris‐buffered saline containing Tween 20 and incubated with secondary antibody for 1 h at room temperature. After washing, the membranes were treated with ECL prime detection reagent (Cytiva, Tokyo, Japan). The bands were visualized using a ChemiDoc XRS System (Bio‐Rad, Hercules, CA, USA).

### Histological analysis

Lung specimens were harvested 7 days after the last administration of *F. nucleatum*, fixed with 4% paraformaldehyde, embedded in paraffin, sectioned, and mounted onto glass slides. To assess the mucin production and goblet cell counts, the slides were stained with Alcian blue/periodic acid‐Schiff (AB/PAS). Goblet cells were counted as described previously [29]. Briefly, two hundred cells were counted, and the number of goblet cells was divided by the total number of cells to calculate the percentage of goblet cells. One slide per animal, for seven animals per group, was assessed. For immunohistochemical analysis, the slides were incubated with anti‐mucin 5AC (MUC5AC) antibody (1 : 50; Abcam Plc, Cambridge, UK) overnight. The samples were then incubated with a secondary antibody for 30 min. The immunolabeling was visualized using DAB substrate, and the staining reaction was observed with a light microscope.

### Statistical analysis

All data are expressed as mean ± SEM. The data were analyzed using one‐way ANOVA with Tukey’s *post hoc* analysis using kaleidagraph (Synergy Software, Reading, PA, USA). Differences were considered significant at *P* < 0.05.

## Results

### Recruitment of inflammatory cells into the bronchoalveolar space

**Fig. 1:**
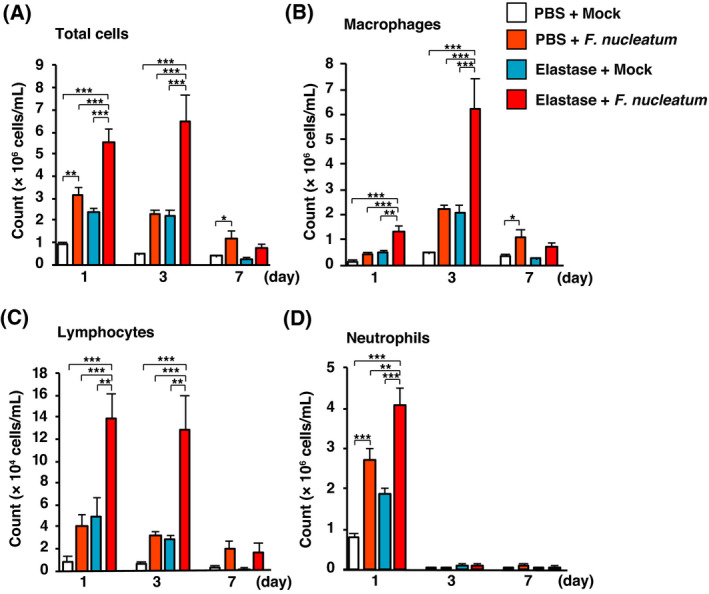
Elastase‐treated mice administered *F. nucleatum* showed altered BALF inflammatory cell profiles. BAL was performed on elastase‐ and PBS‐treated mice administered *F. nucleatum* or the vehicle (Mock). The numbers of total cells (A), macrophages (B), lymphocytes (C), and neutrophils (D) are shown for one, three, and seven days after the administration of *F. nucleatum*. The values are presented as the mean ± SEM, *n* = 7. *P < 0.05; **P < 0.01; ***P < 0.001. Statistical significance was evaluated using one‐way ANOVA with Tukey’s *post hoc* analysis (A–D).

To evaluate whether intratracheal administration of *F. nucleatum* caused a significant increase in the number of inflammatory cells in the elastase‐treated mice, we analyzed the number of inflammatory cells in the BALF from all groups one, three, and seven days after the final administration of *F. nucleatum*. At days 1 and 3, the total cell number, number of macrophages, and number of lymphocytes in the elastase‐treated mice dosed with *F. nucleatum* was significantly higher than in the control mice (Fig. 1A–C). One day after the administration, the accumulation of neutrophils in the BALF was significantly higher in the elastase‐treated mice given *F. nucleatum* than in the other mice (Fig. 1D). Seven days after administration, most of the inflammatory cells in the BALF had disappeared. These results suggest that the administration of *F. nucleatum* to the elastase‐treated mice produced augmented inflammatory responses, similar to those seen in patients with COPD exacerbations [5].

### Administration of *F. nucleatum* enhances cytokine mRNA levels in elastase‐treated mice

**Fig. 2:**
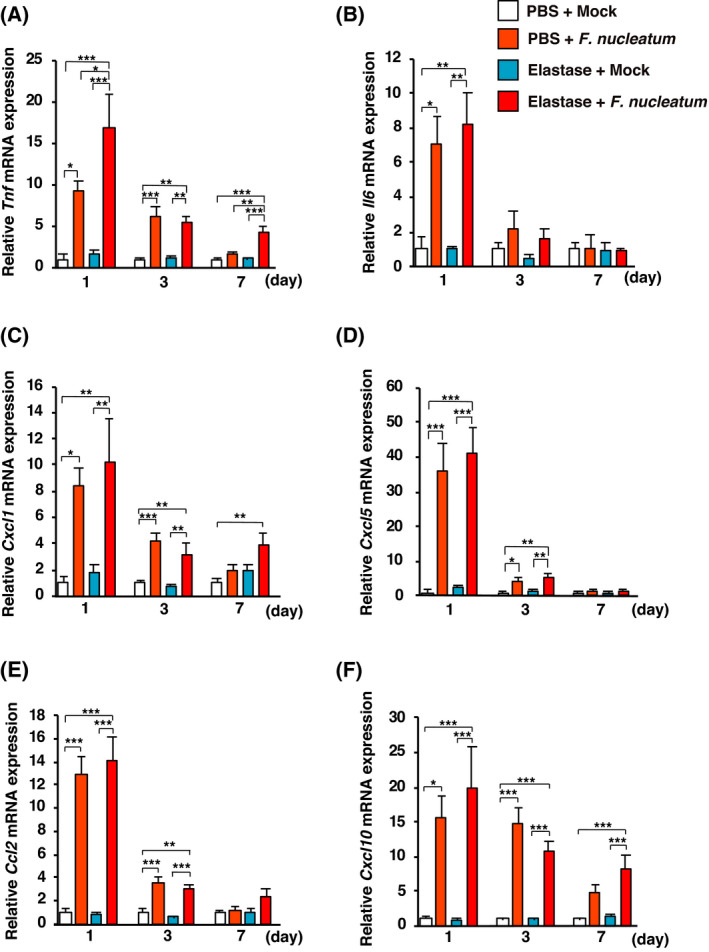
Effects of *F. nucleatum* on the expression of proinflammatory cytokine genes in lungs of elastase‐treated mice. Elastase‐ and PBS‐treated mice were administered *F. nucleatum* or the vehicle (Mock). The lungs were harvested at 1, 3, or 7 days after the administration of *F. nucleatum*. (A) *Tnf*, (B) *Il6*, (C) *Cxcl1*, (D) *Cxcl5*, (E) *Ccl2*, and (F) *Cxcl10* mRNA expression in the lungs was determined using real‐time RT‐PCR. The values are presented as the mean ± SEM, *n* = 7. *P < 0.05; **P < 0.01; ***P < 0.001. Statistical significance was evaluated using one‐way ANOVA with Tukey’s *post hoc* analysis (A–F).

We investigated whether the intratracheal administration of *F. nucleatum* affected the mRNA expression of proinflammatory cytokines and chemokines, including *Tnf*, *Il6*, *Cxcl1*, *Cxcl5*, *Ccl2*, and *Cxcl10*, in the lungs of elastase‐treated mice. The level of *Tnf* mRNA was significantly higher in lungs from elastase‐treated mice given *F. nucleatum* than in the other groups one and seven days after the last administration of *F. nucleatum* (Fig. 2A). The levels of *Il6*, *Cxcl1*, *Cxcl5*, *Ccl2*, and *Cxcl10* mRNA increased following *F. nucleatum* administration to both elastase‐ and PBS‐treated mice (Fig. 2B–F). These results suggest that *F. nucleatum* induced inflammatory responses in the mouse lungs.

### *F. nucleatum* administration enhanced MMP12 and perforin in elastase‐treated mice

**Fig. 3:**
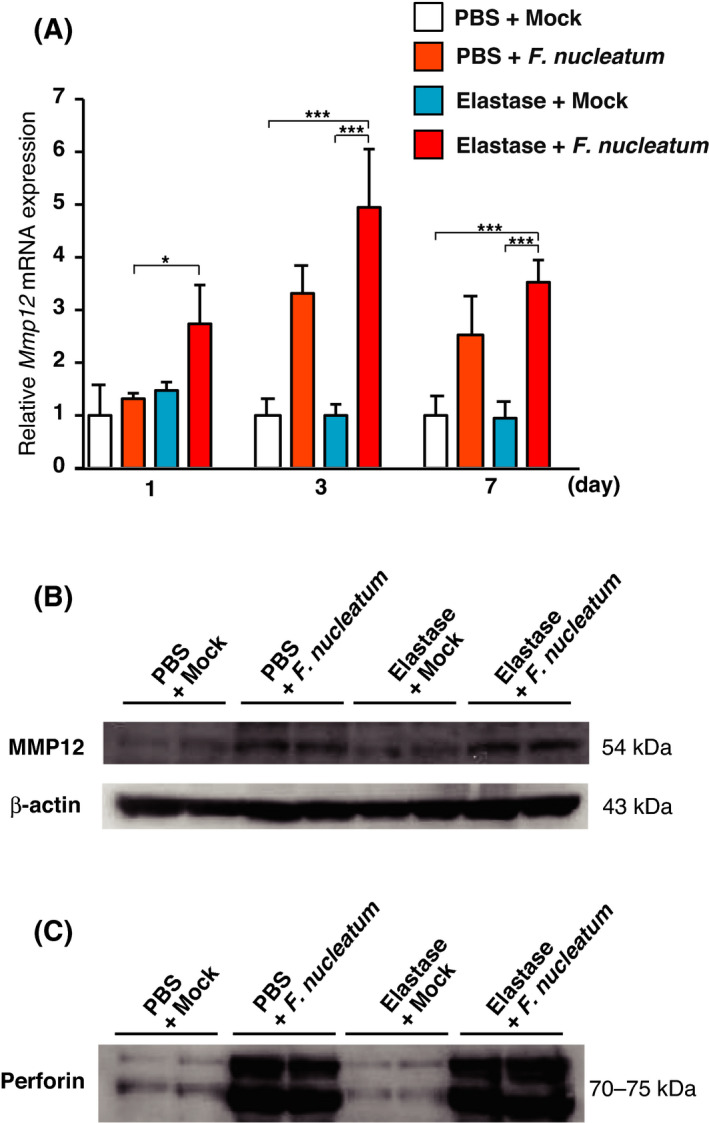
Effects of *F. nucleatum* on the production of MMP12 and perforin in the lungs of elastase‐treated mice. Elastase‐ and PBS‐treated mice were administered either *F. nucleatum* or the vehicle (Mock). (A) The lungs were harvested at one, three, or seven days after the administration of *F. nucleatum*. *Mmp12* mRNA expression in the lungs was determined using real‐time RT‐PCR. The values are presented as the mean ± SEM, *n* = 7. *P < 0.05; ***P < 0.001. Statistical significance was evaluated using one‐way ANOVA with Tukey’s *post hoc* analysis. (B) The lungs were harvested three days after the administration of *F. nucleatum*. MMP12 protein expression was detected using western blotting of lung lysates. (C) BALF was harvested three days after exposure to *F. nucleatum*. Perforin protein expression was detected using western blotting of BALF.

MMP12 degrades the alveolar wall matrix [30] and perforin could induce apoptosis of the alveolar wall [31]. We therefore examined whether intratracheal administration of *F. nucleatum* affected the expression of MMP12 and perforin in lungs from elastase‐treated mice. *Mmp12* mRNA expression was elevated 1, 3, and 7 days after the last *F. nucleatum* administration in elastase‐treated mice (Fig. 3A). The levels of MMP12 protein were also increased in the lungs of elastase‐ and PBS‐treated mice administered *F. nucleatum* (Fig. 3B). We evaluated the secretion of perforin in BALF supernatant 3 days after the administration of *F. nucleatum*. The secretion of perforin was increased by the administration of *F. nucleatum* to both elastase‐ and PBS‐treated mice (Fig. 3C). These data suggest that *F. nucleatum* can induce the expression of factors that contribute to alveolar wall destruction.

### Progression of emphysema was accelerated by *F. nucleatum* in elastase‐treated mice

**Fig. 4:**
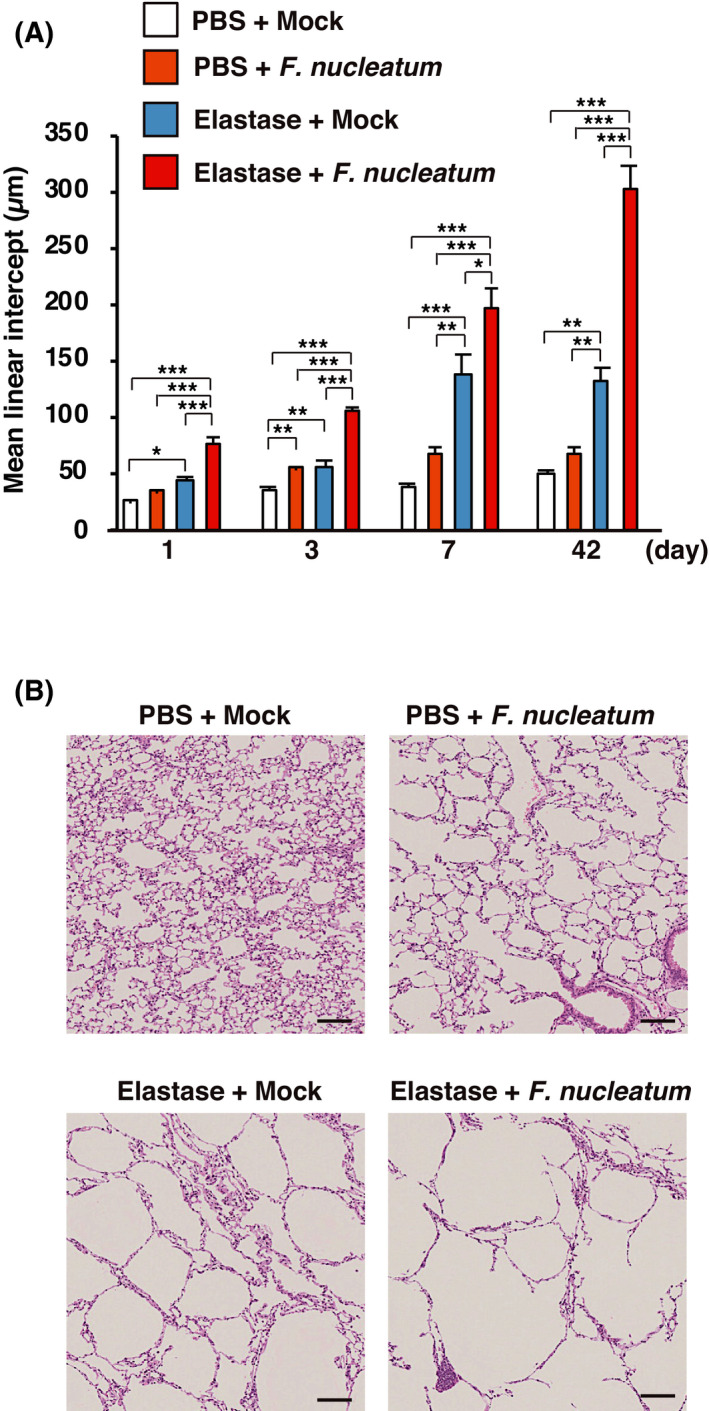
*F. nucleatum* enhanced elastase‐induced alveolar enlargement in elastase‐treated mice. Lung morphology was determined in elastase‐ and PBS‐treated mice at days 1, 3, 7, and 42 after administration of *F. nucleatum* or the vehicle (Mock). (A) Mean linear intercept (Lm) was measured on random sections of the lung (*n* = 7). The data are shown as means ± SEM. *P < 0.05; **P < 0.01; ***P < 0.001. Statistical significance was evaluated using one‐way ANOVA with Tukey’s *post hoc* analysis. (B) Representative lung sections at 42 days after *F. nucleatum* or the vehicle administration stained with hematoxylin and eosin. Scale bar = 100 µm.

To investigate whether *F. nucleatum* contributes to the progression of emphysema in elastase‐treated mice, we assessed the Lm, the mean of the inner diameter of the pulmonary alveoli, and lung specimens 1, 3, 7, and 42 days after the last intratracheal administration of *F. nucleatum* (Fig. 4A). Noticeably, early (1, 3, and 7 days) and late (42 days) harvested lung specimens putatively have a similar pattern of *F. nucleatum* enhanced elastase‐induced alveolar enlargement. Figure 4B shows micrographs of representative lung sections taken from 42 days after the last intratracheal administration of *F. nucleatum* or Mock. We observed obvious enlargement of alveoli in elastase‐treated mice administered *F. nucleatum*. These results indicate that stimulation originated from *F. nucleatum* accelerated the progression of emphysema in the elastase‐treated mice, as is seen in patients with repeated COPD exacerbations [10].

### Administration of *F. nucleatum* enhanced mucin expression in elastase‐treated mice

**Fig. 5:**
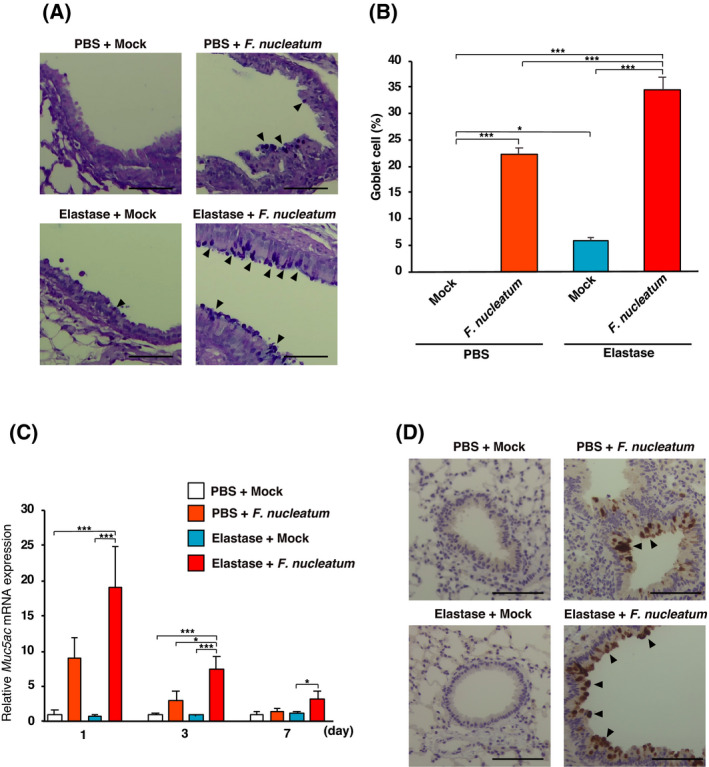
Effects of *F. nucleatum* on goblet cell metaplasia and MUC5AC expression in lungs of elastase‐treated mice. Elastase‐ and PBS‐treated mice were administered either *F. nucleatum* or the vehicle (Mock). The lungs were harvested at 1, 2, or 7 days after the administration of *F. nucleatum*. (A) AB/PAS‐stained bronchi from elastase‐ and PBS‐treated mice seven days after the administration of either *F. nucleatum* or vehicle (Mock). Scale bar = 100 µm. (B) Percentage of goblet cells seven days after the administration of *F. nucleatum*. The values are presented as the mean ± SEM, *n* = 7. *P < 0.05; ***P < 0.001. (C) *Muc5ac* mRNA expression in the lungs determined using real‐time RT‐PCR. The values are presented as the mean ± SEM, *n* = 7. **P < 0.01; ***P < 0.001. (D) Immunohistochemistry for MUC5AC on bronchi from elastase‐ and PBS‐treated mice 7 days after the administration of *F. nucleatum* or Mock. Scale bar = 100 µm. Statistical significance was evaluated using one‐way ANOVA with Tukey’s *post hoc* analysis (B and C).

Goblet cell metaplasia contributes to the hypersecretion of mucus in the airway, which has been associated with accelerated decline in lung function and progression of COPD [11, 32]. Therefore, we examined whether the intratracheal administration of *F. nucleatum* affects goblet cell metaplasia and the expression of MUC5AC, the major respiratory mucin [33], in the lungs of elastase‐treated mice. As shown in Fig. 5A, goblet cell metaplasia was observed in mice administered *F. nucleatum*. In particular, the quantification of AB/PAS staining confirmed that the number of goblet cells was significantly increased in elastase‐treated mice administered *F. nucleatum* (Fig. 5B). We found that the expression of *Muc5ac* mRNA was increased in the lungs of elastase‐treated mice administered *F. nucleatum* compared with the other groups (Fig. 5C). As shown using immunohistochemical staining for MUC5AC (Fig 5D), the MUC5AC protein accumulated along the epithelial cells of the airway of elastase‐treated mice administered *F. nucleatum* compared with PBS‐treated mice administered *F. nucleatum*. These results imply that *F. nucleatum* was associated with the mucus hypersecretion through goblet cell metaplasia and expression of MUC5AC.

## Discussion

Periodontal disease has been shown to exacerbate pulmonary disease, including COPD [16, 34, 35]. Various mechanisms that produce this effect have been proposed, including the aspiration of oral bacteria [36]. COPD and periodontal disease share a similar mechanism of tissue destruction, characterized by inflammatory responses and release of proteolytic enzymes, resulting in the destruction of the pulmonary alveoli or the periodontal tissues [37, 38]. Periodontal pathogens, including *F. nucleatum*, are probably associated with the exacerbation of COPD [19, 20, 39]. However, little is known about how periodontal pathogens affect COPD exacerbations *in vivo*. In this study, we attempted to elucidate the effect of *F. nucleatum* on mice with elastase‐induced emphysema. The intratracheal administration of *F. nucleatum* to the lungs of elastase‐treated mice exacerbated inflammatory responses, leading to increased numbers of inflammatory cells and increased levels of proinflammatory cytokines. The administration of *F. nucleatum* enhanced the production of factors that contribute to alveolar wall destruction and accelerated the progression of emphysema. *F. nucleatum* also induced the hypersecretion of mucus in the airways. These observations suggest that *F. nucleatum* plays a role in the exacerbation of COPD.

One and three days after the administration of *F. nucleatum*, the number of total cells in the BALF was significantly higher in elastase‐treated mice administered *F. nucleatum* than in any of the other treatment groups. Elastase‐treated mice experienced a strong recruitment of neutrophils, macrophages, and lymphocytes to the lung in response to *F. nucleatum*. The severity of COPD is related to the degree to which the lung tissue is infiltrated by inflammatory cells such as neutrophils, macrophages, and lymphocytes [40]. Neutrophils have the capacity to induce tissue damage through the release of serine proteases such as elastase [41, 42]. Alveolar macrophages from COPD patients release more inflammatory mediators, such as TNF‐α and IL‐6, than macrophages from control subjects, and these inflammatory mediators play an important role in the pathophysiology of COPD [42]. There is an increase in the number of T lymphocytes in the lung parenchyma and airways of patients with COPD [41, 42] and increased release of perforin from CD8^+^ T lymphocytes [42]. These observations, together with our findings, suggest that *F. nucleatum*‐induced recruitment of inflammatory cells plays an essential role in the exacerbation of COPD.

Cytokines and chemokines play critical roles in many pathobiological processes in COPD, including the infiltration of inflammatory cells into lung tissue, exacerbation, and emphysema [7, 43, 44]. Inflammatory cytokines induced by pneumonia‐related bacteria, such as *Haemophilus influenzae* and *Streptococcus pneumoniae*, are important in COPD exacerbation [4, 42, 43], and TNF‐α and IL‐6 levels are increased in sputum during COPD exacerbations [7, 44]. The current study indicated that the administration of *F. nucleatum* induced the expression of the *Tnf* and *Il6* genes in elastase‐treated mouse lungs. Our previous studies demonstrated that *F. nucleatum* is a potent stimulator of TNF‐α and IL‐6 in human respiratory epithelial cells [25, 26, 45]. In the current study, although the expression of chemokine genes in the lungs of elastase‐treated mice administered *F. nucleatum* was not significantly different from that in the lungs of PBS‐treated mice administered *F. nucleatum*, *F. nucleatum* caused a significant increase in the number of inflammatory cells in the BALF, which were enhanced in the lungs of elastase‐treated mice. This finding suggests that elastase‐treated lungs lead to a more active cellular entry compared with PBS‐treated lungs. Hence, it appears that the *F. nucleatum*‐induced recruitment of cytokines and chemokines plays an important role in the exacerbation of COPD.

Emphysema, destruction of the alveolar wall, is a major pathological feature of the lungs of individuals with COPD [3]. Emphysema progression is accelerated by exacerbations in patients with COPD [10]. In the current study, the emphysema in elastase‐treated mice administered with *F. nucleatum* progressed to a greater extent than in mice treated with *F. nucleatum* alone or those pretreated with elastase alone. Moreover and quite surprisingly, the progression significantly occurred as early as day 1 which suggests that if continuous aspiration is occurring among COPD patients with periodontal disease, COPD can be exacerbated quite easily in these patients due to aspiration of saliva which has high *F. nucleatum* levels. Administration of *F. nucleatum* induced the expression of the alveolar wall destruction factors MMP12 and perforin in mice. MMP12, which is secreted by macrophages, and perforin, which is secreted by CD8^+^ T lymphocytes, are believed to promote the degradation of the alveolar wall, contributing to the development of emphysematous lesions [30, 31, 46]. Our observations, together with previous findings, suggest that *F. nucleatum* has the potential to increase the progression of emphysema by inducing the release of MMP12 and perforin in patients with COPD.

Mucus hypersecretion in patients with COPD has been associated with compromising lung function, contributing to the severity of the disease, and with increased mortality [47, 48]. The administration of *F. nucleatum* increased goblet cell metaplasia and MUC5AC expression, which were more strongly enhanced in elastase‐treated mice. MUC5AC is partially induced by TNF‐α in the epithelial cells of the airway [11, 49]. The expression of *Tnf* was significantly higher in elastase‐treated mice administered *F. nucleatum* than in PBS‐treated mice administered *F. nucleatum*. TNF‐α expression may therefore contribute to *F. nucleatum*‐induced MUC5AC expression. Supernatants from *F. nucleatum* cultures promote the upregulation of MUC5AC expression in human airway epithelial cell lines [50]. Our previous study showed that MUC5AC expression was induced by *P. gingivalis* gingipain in human airway epithelial cell lines and primary cells [51]. These observations, together with our findings, suggest that major periodontal pathogens are potent inducers of the expression of mucin in patients with COPD.

The involvement of periodontal disease in COPD has been suggested by the results of many recent studies. Poor periodontal health is associated with COPD occurrence [15, 52], COPD exacerbation [16, 53]. Periodontal treatment in COPD patients with chronic periodontitis reduces the frequency of COPD exacerbations [54, 55]. The levels of antibody against *F. nucleatum* are markedly elevated in the sputum of patients with COPD exacerbations [20], and the number of bacteria of the genus *Fusobacterium* is higher in the sputum of patients with severe COPD [24]. In patients with COPD, an abnormal swallowing reflex is associated with the frequency of COPD exacerbation [56, 57]. The salivary concentration of *F. nucleatum* is increased in patients with periodontal diseases [58]. Consequently, aspiration of the saliva of periodontal disease, including *F. nucleatum*, may play a role in the induction of COPD exacerbations. It is therefore tempting to speculate that healthy periodontal conditions and good oral hygiene may prevent or reduce COPD exacerbations.

The major limitation of our study was that we used only heat‐killed whole bacterial cells for the investigation of the effects of *F. nucleatum* on elastase‐treated mice. Our findings suggest that because infection was not necessarily required for the exacerbations, these are caused by a virulence factor from *F. nucleatum*. *F. nucleatum* possesses virulence factors including adhesins, LPS, serine protease, and butyrate [19, 59]. In particular, fusobacterial adhesins, such as FadA, Fap2, and RadD, facilitate adhesion and invasion to host cells, and the triggering of host immune responses [19, 59]. These findings combined with our observations may provide a new insight for COPD as a non‐infectious disease which can be exacerbated by stimulation of *F. nucleatum* aspirated into the lower respiratory tracts. In addition, the amount of *F. nucleatum* that we used in the present study should be estimated to be adequate when referring to the facts that *F. nucleatum* is present in saliva at 8 × 10^4^–3 × 10^7^ CFU·mL^−1^ in individuals with healthy periodontium and that the number of *F. nucleatum* in saliva of patients with periodontal disease is 100–1000 times higher than that of those with healthy periodontium [58, 60]. Although we were able to link *F. nucleatum* with COPD exacerbations, further studies that focus on *F. nucleatum* virulence factors are needed to identify the details of the mechanism of *F. nucleatum*‐induced COPD exacerbation.

Periodontal disease and COPD are spreading worldwide. COPD is associated with the development of a range of diseases, such as ischemic heart disease, diabetes, osteoporosis, and lung cancer [3, 61]. Although periodontal disease has recently been implicated as a risk factor for COPD exacerbations, the causal relationship remains poorly understood. This study provides evidence suggesting that *F. nucleatum*, as one of the key periodontopathic bacteria that are aspirated into the lung, could cause exacerbation of COPD. Periodontal treatment is effective in reducing the frequency of COPD exacerbation in patients with COPD and periodontal disease [17], and it is therefore considered that aspirated periodontopathic bacteria may be a risk factor for COPD exacerbation.

## Conflict of interest

The authors declare no conflict of interest.

## Author contributions

RS and NK designed the experiments, analyzed the data, and wrote the paper. TK and YY contributed to the discussion, analyzed data, and reviewed drafts of the manuscript. KI was responsible for the study concept and design, analysis of results and manuscript writing. All authors have read and agreed to the published version of the manuscript.

## Data Availability

The data that support the findings of this study are available from the corresponding author upon reasonable request.
